# Neoadjuvant anthracycline-containing vs. anthracycline-free chemotherapy with dual HER2 blockade in HER2-Positive breast cancer: a Systematic Review and meta-analysis

**DOI:** 10.1515/med-2026-1525

**Published:** 2026-09-10

**Authors:** Wenwen Bai, Qingyu Wang, Zhiguo Zhou, Xiaoyan Lv, Yajing Wu, Yuzhi Song, Ruohui Zhang, Rui Zhang, Yunjie Cheng, Xinyi Li, Jun Wang

**Affiliations:** Department of Radiation Oncology, The Fourth Hospital of Hebei Medical University, Shijiazhuang, China

**Keywords:** breast neoplasms, neoadjuvant therapy, dual HER2 blockade, anthracyclines

## Abstract

**Objectives:**

Neoadjuvant chemotherapy with dual HER2 blockade is the standard of care for early-stage HER2-positive breast cancer. However, the role of anthracyclines remains controversial. This meta-analysis aimed to evaluate the efficacy and safety of neoadjuvant dual HER2 blockade combined with anthracycline-containing vs. anthracycline-free chemotherapy.

**Methods:**

A literature search was conducted across PubMed, EMBASE, and the Cochrane Library. Eligible studies included patients with resectable stage I-III HER2-positive breast cancer receiving neoadjuvant dual HER2 blockade with either an anthracycline-containing (A-based) or anthracycline-free (non-A) chemotherapy regimen. Standard meta-analytic methods using a random-effects or fixed-effect model were applied according to heterogeneity (I^2^).

**Results:**

Seven articles met the inclusion criteria. The addition of anthracyclines to dual HER2 blockade therapy (DHBT) did not significantly improve the pCR rate (OR, 1.07; 95 % CI, 0.81–1.43; p=0.62), composite DFS/EFS/PFS (HR, 0.50; 95 % CI, 0.18–1.38; p=0.18), or overall survival (OS) (HR, 0.77; 95 % CI, 0.39–1.51; p=0.45). The incidence of cardiac adverse events did not differ significantly between the A-based and non-A groups (OR, 0.96; 95 % CI, 0.31–2.91; p=0.94). No significant difference was observed in the incidence of LVEF decline ≥10 % from baseline to <50 % between the A-based and non-A groups (OR, 1.47; 95 % CI, 0.79–2.77; p=0.23). Notably, the non-A group had a significantly higher incidence of grade ≥3 diarrhea (OR, 0.60, favoring the A-based group; 95 % CI, 0.38–0.95; p=0.03).

**Conclusions:**

In resectable HER2-positive breast cancer, neoadjuvant DHBT without anthracyclines achieves pCR rates non-inferior to A-based regimens, with no early survival detriment observed. While short-term cardiotoxicity is unremarkable, grade ≥3 diarrhea occurs more frequently with non-A approaches.

## Introduction

Human epidermal growth factor receptor 2 (HER2)-positive breast cancer accounts for approximately 20–25 % of all breast cancers and is a highly aggressive subtype that has traditionally been associated with poor prognosis [1]. This characteristic has driven the continuous innovation of treatment strategies. The introduction of anti-HER2 targeted therapy, especially the dual HER2 blockade therapy (DHBT) combining trastuzumab and pertuzumab with chemotherapy in the neoadjuvant setting, has markedly increased pathological complete response (pCR) rates and improved survival, establishing it as a standard treatment for this breast cancer subtype [2], [3], [4], [5], [6], [7], [8]. In the era of dual HER2 blockade, the optimal chemotherapy backbone – specifically, whether anthracycline-containing (A-based) regimens remain essential – continues to be debated. This controversy centers on balancing efficacy and safety: while anthracyclines are established as highly active agents, their cardiotoxic mechanisms are well characterized [9]. Moreover, combining anthracyclines with anti-HER2 therapies may potentiate cardiac injury, heightening the risk of cardiotoxicity [10]. Thus, identifying strategies that preserve efficacy while minimizing toxicity represents a key priority in optimizing neoadjuvant therapy for HER2-positive breast cancer.

The TRAIN-2 trial was a pivotal turning point in this debate. In patients with stage II-III HER2-positive breast cancer receiving trastuzumab plus pertuzumab, omission of anthracyclines from the chemotherapy backbone produced similar pCR and 3-year event-free and overall survival outcomes while reducing several clinically important toxicities [10], 11]. This evidence weakened the assumption that anthracyclines are indispensable in the neoadjuvant setting and contributed to guideline-level preference for platinum-taxane-based regimens such as TCbHP, with anthracycline-containing regimens retained as alternatives [12].

Nevertheless, subsequent evidence has kept the debate open. KRISTINE showed that a conventional chemotherapy-containing TCHP regimen achieved a higher pCR rate and fewer preoperative locoregional progression events than T-DM1 plus pertuzumab, supporting the continued relevance of chemotherapy with dual blockade rather than a fully chemotherapy-free approach [13]. TRYPHAENA demonstrated that both anthracycline-containing and anthracycline-free dual-blockade regimens can achieve substantial pCR rates with manageable short-term cardiac safety [14], 15]. In addition, retrospective data in HER2-positive inflammatory breast cancer suggested that anthracycline-containing neoadjuvant therapy may improve locoregional control and EFS despite similar pCR rates [16], and ABCSG-52/ATHENE reported a high pCR rate with an epirubicin-containing dual-blockade regimen without an obvious cardiac safety signal [17]. These findings create tension with the current guideline trend favoring anthracycline-free strategies, underscoring the need to re-evaluate the role of anthracyclines in the dual HER2 blockade era within a higher-level, evidence-based framework.

A recent meta-analysis addressed the anthracycline question, but it predominantly included studies using single-agent HER2 blockade and incorporated retrospective data, introducing potential bias and limiting applicability to the current standard of care [18]. The novelty of our study lies in its exclusive focus on neoadjuvant DHBT with trastuzumab plus pertuzumab and its inclusion of prospective studies only. This meta-analysis aimed to provide an updated synthesis of the evidence by systematically evaluating the efficacy and safety of combining dual HER2 blockade with A-based vs. non-A chemotherapy, thereby informing clinical practice and future trial design.

## Materials and methods

### Systematic Review and search strategy

This study was registered in the PROSPERO database (No. CRD420251053996) and followed the Preferred Reporting Items for Systematic Reviews and Meta-Analyses (PRISMA) and Meta-Analyses of Observational Studies in Epidemiology (MOOSE) guidelines (Supplementary Tables S4 and S5). A systematic literature search was performed in the Cochrane Library, PubMed, and EMBASE databases for English-language articles published from January 1, 2010, to February 6, 2026. Detailed search strategies are provided in Supplementary Tables S1–S3.

### Eligibility criteria

Inclusion criteria were defined using the PICOD framework:

– **P (Patients):** Patients with histopathologically confirmed, resectable stage I-III HER2-positive breast cancer.
– **I (Intervention):** Neoadjuvant therapy with dual HER2 blockade (Trastuzumab + Pertuzumab) combined with A-based chemotherapy regimen.
– **C (Comparison):** Neoadjuvant therapy with dual HER2 blockade (Trastuzumab + Pertuzumab) combined with non-A chemotherapy regimen.
– **O (Outcomes):** Pathological complete response (pCR), disease-free survival (DFS), event-free survival (EFS), progression-free survival (PFS), overall survival (OS), cardiac adverse events (CAEs), and other treatment-related adverse events (TRAEs) of grade ≥3.
– **D (Design):** Prospective studies, including randomized controlled trials (RCTs), non-randomized controlled studies, and observational cohort studies.

Exclusion criteria included: (1) receipt of other treatments before neoadjuvant therapy; (2) dual HER2 blockade not based on trastuzumab plus pertuzumab; (3) reviews, case reports, letters, or book chapters; (4) non-human studies; (5) small sample size (n≤15); and (6) studies with incomplete data for analysis. If a trial had been updated, we generally included the publication with the most complete data. However, when different publications from the same trial reported different outcomes, publications from different years could be included for endpoint-specific analyses; for DFS/EFS/PFS, OS, and CAEs, the most recently published data were prioritized.

### Data extraction and quality assessment

Two authors (W.B. and R.Z.) independently extracted data, and two others (Q.W. and X.L.) validated the extraction. If important data in the included studies were missing, authors of the original publications were contacted. Discrepancies were resolved by consensus (inter-rater agreement measured by Cohen’s kappa coefficient: κ=0.80; p<0.001) [19]. Extracted data included study characteristics, baseline demographics, treatment regimens, and all specified outcomes. When individual studies reported long-term outcomes using different but related disease-control endpoints (DFS, EFS, or PFS), these were pooled as a composite disease-control endpoint because each captures recurrence, progression, invasive events, or death after neoadjuvant therapy and because endpoint-specific data were sparse. We recognized that these endpoints are not identical; therefore, sensitivity analyses were performed by omitting individual studies and, where feasible, by examining the influence of endpoint type. When a survival rate was 100 % in one group, a continuity correction was applied to permit statistical estimation.

Methodological quality was independently assessed by two authors. For RCTs, the Revised Cochrane risk-of-bias tool (RoB2) was used (Supplementary Figures S1–S2). For non-randomized studies, the Methodological Index for Non-Randomized Studies (MINORS) was applied [20] (Supplementary Table S6). For observational studies, the Newcastle-Ottawa Scale (NOS) was used (Supplementary Table S7).

### Statistical analysis

The meta-analysis was performed using Review Manager (RevMan, version 5.4.1). The log-transformed odds ratio (OR) or hazard ratio (HR) and their standard errors were calculated using Stata, version 19.0 (StataNow MP). Statistical heterogeneity among studies was assessed using the chi-squared test and the I^2^ statistic [21]. An I^2^ value <50 % indicated low heterogeneity, and a fixed-effect model (Mantel–Haenszel) was used; otherwise, a random-effects model was employed.

Dichotomous outcomes (pCR, adverse events) were analyzed using ORs with 95 % confidence intervals (CIs). Time-to-event data (DFS/EFS/PFS, OS) were analyzed using HRs with 95 % CIs and pooled using the inverse variance method. A leave-one-out sensitivity analysis was performed for outcomes with an I^2^ statistic ≥30 % to evaluate robustness. For the composite DFS/EFS/PFS endpoint, sensitivity analyses also examined whether results were driven by any single endpoint type where feasible. Publication bias was evaluated using funnel plots.

## Results

### Literature review and study characteristics

**Figure 1:**
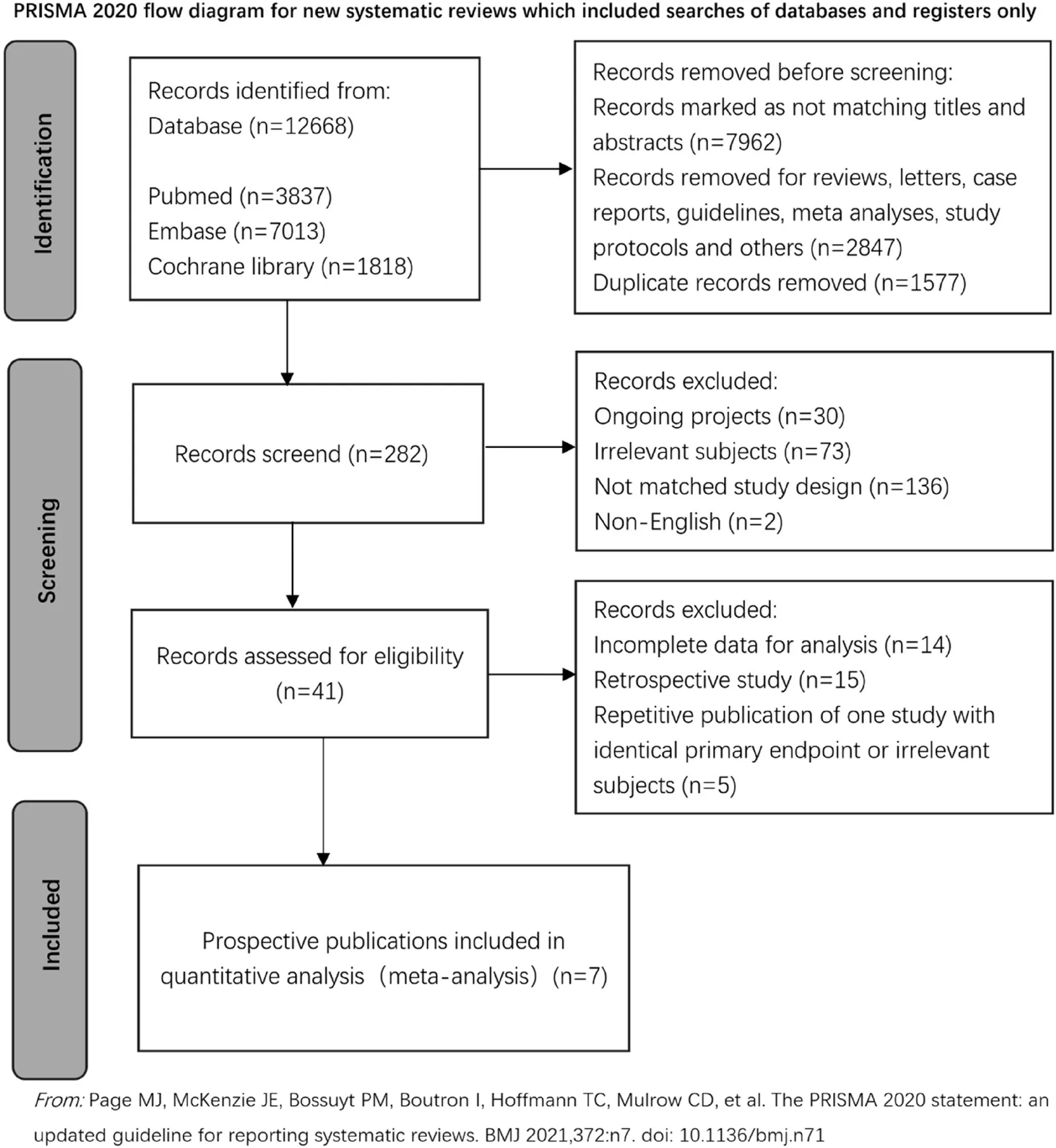
Study selection diagram of this meta-analysis. PRISMA, preferred reporting items for systematic reviews and meta‐analyses.

The search yielded seven eligible articles representing five unique trials that met the inclusion criteria (Figure 1). These comprised two RCTs, one prospective non-randomized trial, and two prospective observational studies. Two publications from TRYPHAENA and two from TRAIN-2 reported different outcomes from the same underlying trials; endpoint-level contributions are shown in Table 1 [10], 11], 14], 15], [22], [23], [24]. Across the trials, the dual HER2 blockade regimen exclusively consisted of trastuzumab and pertuzumab. The definitions of pCR, DFS, EFS, PFS, OS and CAEs in different studies are summarized in Supplementary Table S8 [10], 11], 14], 15], [22], [23], [24]. Additionally, the details regarding differences in chemotherapy backbone, supportive care strategies, and toxicity assessment methods across studies are shown in Supplementary Table S9 [10], 11], 14], 15], [22], [23], [24].

**Table 1:** General information from the included studies.

Trial/year	Trial type	Arms	Clinical stage	No. of patients	Median age (years)	Median follow-up (months)	Outcomes	Endpoint(s) contributed to meta-analysis
TRYPHAENA 2013 [14]	Randomized, controlled trial, phase 2	Group A: (FEC + H + P) × 3 cycles→ (T^a^ + H + P) × 3 cycles	Operable (T2-3, N0-1, M0), locally advanced (T2-3, N2 or N3, M0; T4a-c, any N, M0), inflammatory (T4d, any N, M0)	Group A: 73	Group A: 49 (27–77)	–	Safety, pCR, clinical response rate, time to clinical response, BCS	pCR, neutropenia, febrile neutropenia, anemia, thrombocytopenia, diarrhea, vomiting.
		Group B: FEC × 3→(T^a^ + H + P) × 3 cycles		Group B: 75	Group B: 49 (24–75)			
		Group C: (TCH + P) × 6 cycles		Group C: 77	Group C: 50 (30–81)			
TRYPHAENA 2018 [15]	Randomized, controlled trial, phase 2	Group A: (FEC + H + P) × 3 cycles→ (T^a^ + H + P) × 3 cycles	Operable (T2-3, N0-1, M0), locally advanced (T2-3, N2 or N3, M0; T4a-c, any N, M0), inflammatory (T4d, any N, M0)	Group A: 73	Group A: 49 (27–77)	Group A:61.1	Cardiac safety, pCR, 3Y-DFS,3Y-PFS, 3Y-OS	DFS, OS, CAEs, LVEF decline ≥10 % points from baseline to <50 %.
		Group B: FEC × 3→(T^a^ + H + P) × 3 cycles		Group B: 75	Group B: 49 (24–75)	Group B:59.4		
		Group C: (TCH + P) × 6 cycles		Group C: 77	Group C: 50 (30–81)	Group C:60.9		
TRAIN-2 2018 [11]	Randomized, controlled trial, phase 3	Group A: (H + P + FEC) × 3→(H + P + TC) × 3 cycles	II–III	Group A: 220	Group A: 48 (43–56)	19 (16–23)	pCR, safety	pCR, neutropenia, febrile neutropenia, anemia, thrombocytopenia, diarrhea, vomiting
		Group B: (H + P + TC) × 9 cycles		Group B: 218	Group B: 49 (43–55)			
TRAIN-2 2021 [10]	Randomized, controlled trial, phase 3	Group A: (H + P + FEC) × 3→(H + P + TC) × 3 cycles Group B: (H + P + TC) × 9 cycles	II–III	Group A: 220	Group A: 48 (43–56)	48.8 (44.1–55.2)	3Y-EFS, 3Y-DFS, 3Y-OS, safety	EFS, OS, CAEs, LVEF decline ≥10 % points from baseline to <50 %.
				Group B: 218	Group B: 49 (43–55)			
Medina 2023 [22]	Prospective, longitudinal, observational study	Group A: (ddAC-DPT) × 8 cycles	IIA–IIIC	Group A: 58	54 (30–83)	–	pCR, safety	pCR, CAEs, LVEF decline, neutropenia, febrile neutropenia, anemia, thrombocytopenia, diarrhea, vomiting.
		Group B: DPT × 4 cycles		Group B: 29				
NeoTOP 2024 [23]	Multicentre, open-label, non-randomized trial, phase 2	Group A: FEC × 3→DPT × 3 cycles	I–IIA	Group A: 41	Group A: 53 (46–59)	40 (17–69)	pCR, 3Y-DFS,3Y-OS, 5Y-DFS, 5Y-OS, safety	pCR, PFS, OS, neutropenia, febrile neutropenia, anemia, thrombocytopenia, diarrhea, vomiting.
		Group B: (DC + HP) × 6 cycles		Group B: 33	Group B: 46 (41–52)			
Alonso–Romero 2026 [24]	Prospective, multicenter, observational study	Group A: Adriamycin/epirubicin + cyclophosphamide × 4→(paclitaxel/docetaxel + CT-P6 + P) × 4 cycles	II–III	Group A + C: 47	Group A+C: 45 (30–78)	–	pCR, safety, soluble HER2, anti-trastuzumab CT-P6 antibodies, and exploratory response-related modeling approaches	pCR
		Group B: (TC + CT-P6 + P) × 6 cycles		Group B: 55	Group B: 56 (36–74)			
		Group C: (T^a^ + CT-P6 + P) × 3→(epirubicin + CT-P6 + P) × 4 cycles						

H, trastuzumab; P, pertuzumab; FEC, 5-fluorouracil + epirubicin + cyclophosphamide; Ta, docetaxel; TCH, docetaxel + carboplatin + trastuzumab; TC, paclitaxel + carboplatin; ddAC, dose-dense anthracycline-based regimen (doxorubicin 60 mg/m^2^ + cyclophosphamide 600 mg/m^2^ on day 1 every 14 days); DPT, docetaxel + pertuzumab + trastuzumab; DC, docetaxel + carboplatin; HP, trastuzumab + pertuzumab; CT-P6, trastuzumab-pkrb, a trastuzumab biosimilar; pCR, pathological complete response; Y, year; DFS, disease-free survival; PFS, progression-free survival; OS, overall survival; EFS, event-free survival; BCS, breast-conserving surgery; –, unknown.

### Efficacy outcomes

**Figure 2:**
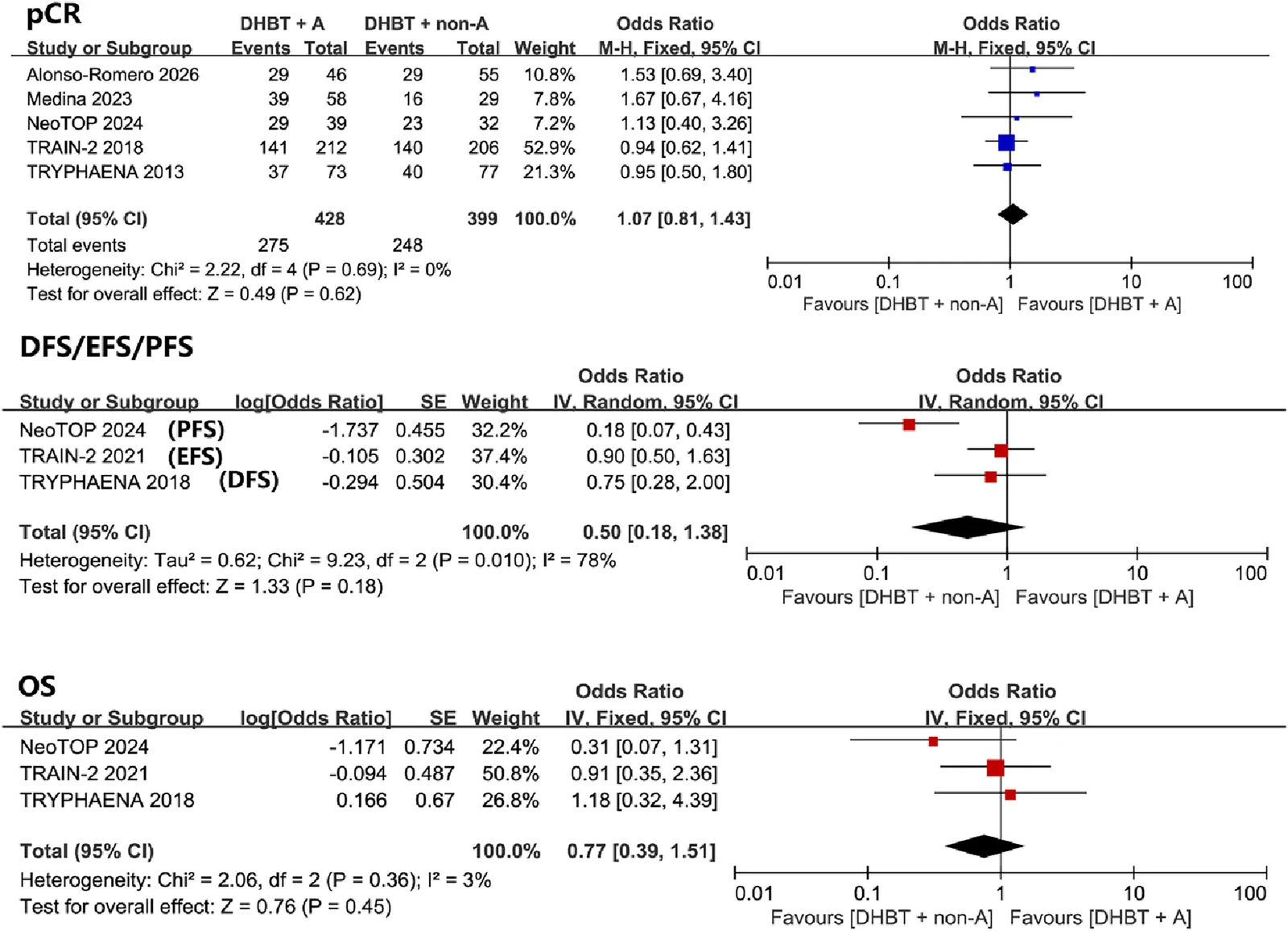
Pooled analyses of pCR, DFS/EFS/PFS, and OS when comparing DHBT + A vs. DHBT + non-A. According to the available data for comparative analysis, pCR was available in five studies, and DFS/EFS/PFS and OS were each available in three studies. CI, indicates confidence interval; df, degrees of freedom; HP, trastuzumab and pertuzumab; A, anthracycline; pCR, pathological complete response; DFS, disease-free survival; EFS, event-free survival; PFS, progression-free survival; OS, overall survival; DHBT + A, dual HER2 blockade treatment + anthracycline-containing chemotherapy; DHBT + non-A, dual HER2 blockade treatment + anthracycline-free chemotherapy.

Data for pCR were available from five studies. Compared with the non-anthracycline group (non-A), the anthracycline-containing group (A-based) was not associated with a higher pCR rate (OR, 1.07; 95 % CI, 0.81–1.43; p=0.62), with no observed heterogeneity (I^2^=0 %) (Figure 2).

DFS, EFS, and PFS were each reported in only a single study; consequently, they were aggregated into a composite DFS/EFS/PFS disease-control endpoint to enable pooled analysis. Three studies were included in the OS analysis. No significant differences were found between the A-based and non-A groups for DFS/EFS/PFS (HR, 0.50; 95 % CI, 0.18–1.38; p=0.18) or OS (HR, 0.77; 95 % CI, 0.39–1.51; p=0.45). Substantial heterogeneity was observed for the composite DFS/EFS/PFS endpoint (I^2^=78 %), whereas heterogeneity was low for OS (I^2^=3 %) (Figure 2).

### Safety and toxicity

**Figure 3:**
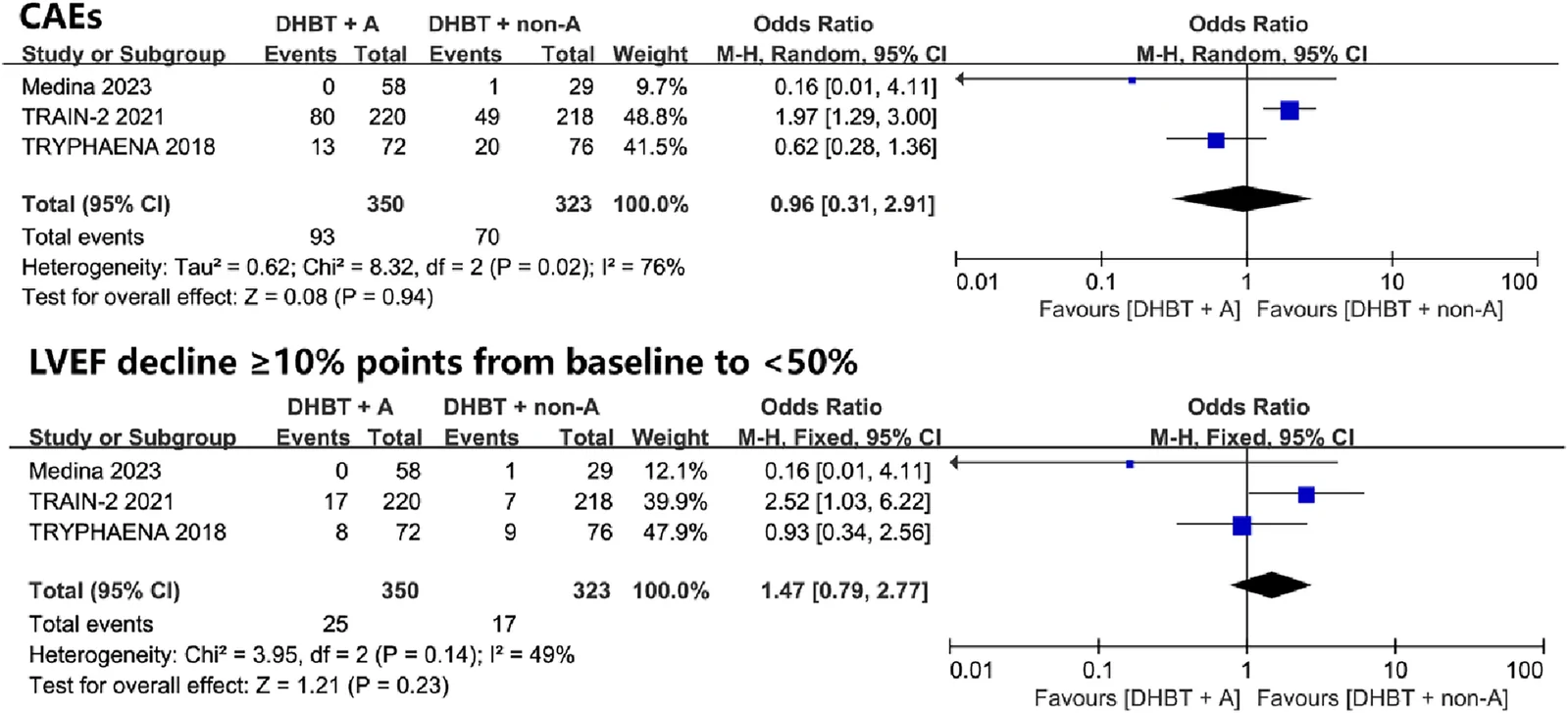
Pooled analyses of CAEs and LVEF decline ≥10 % points from baseline to <50 % when comparing DHBT + A vs. DHBT + non-A. According to the available data for comparative analysis, CAEs and LVEF decline were each available in three studies. CAEs, cardiac adverse events; LVEF, left ventricular ejection fraction.

Three studies reported CAEs. There was no significant difference in the incidence of CAEs between the A-based and non-A groups (OR, 0.96; 95 % CI, 0.31–2.91; p=0.94). No significant difference was observed in the incidence of left ventricular ejection fraction (LVEF) decline ≥10 % from baseline to <50 % (OR, 1.47; 95 % CI, 0.79–2.77; p=0.23) (Figure 3). Heterogeneity was substantial for CAEs (I^2^=76 %) and moderate for LVEF decline (I^2^=49 %).

**Figure 4:**
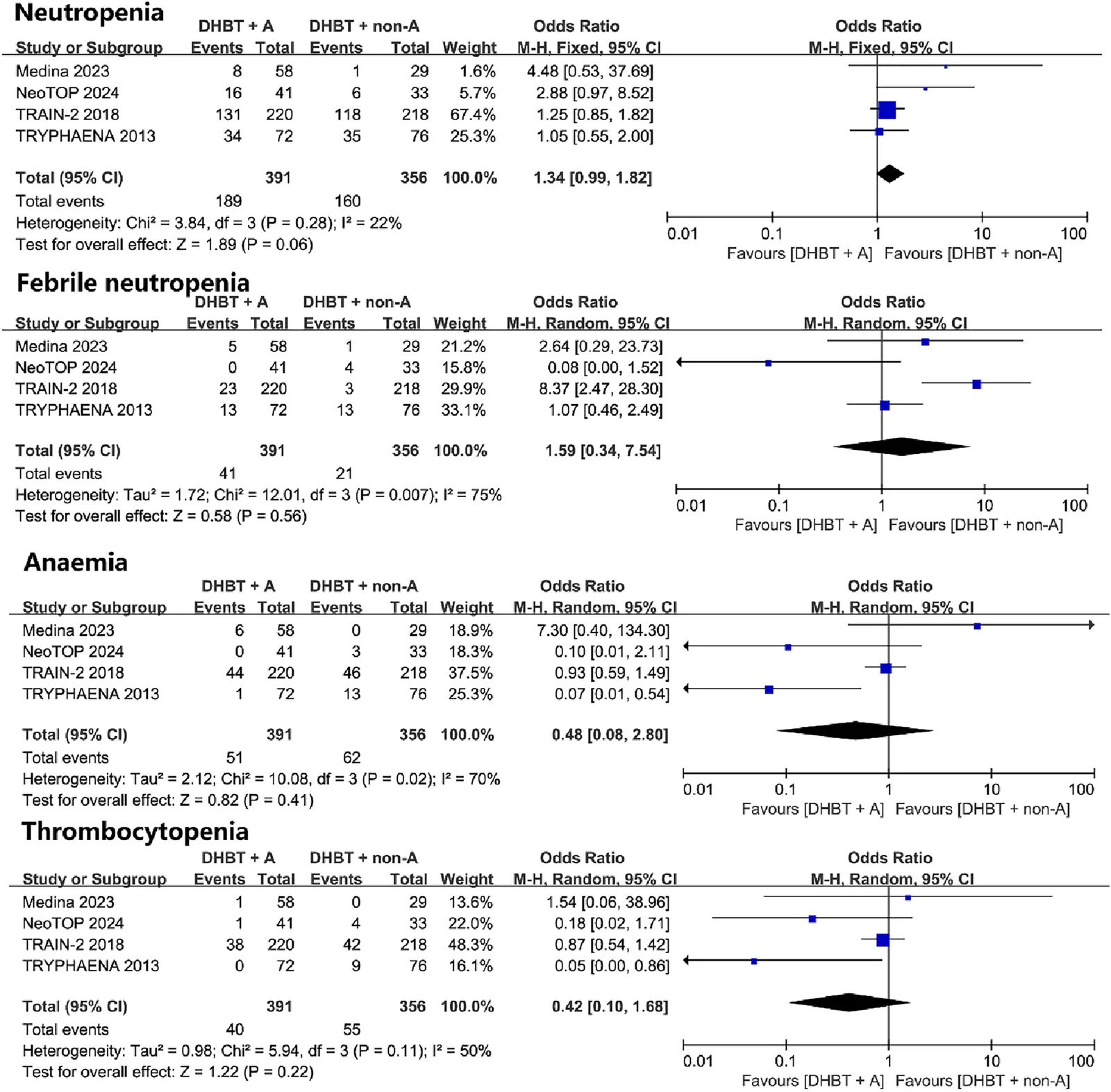
Pooled analyses of grade ≥3 hematological toxicity when comparing DHBT + A vs. DHBT + non-A. According to the available data for comparative analysis, neutropenia, febrile neutropenia, anemia, and thrombocytopenia were each available in four studies.

Regarding grade ≥3 TRAEs, the incidences of hematological toxicities were comparable between the two groups: neutropenia (OR, 1.34; 95 % CI, 0.99–1.82; p=0.06), febrile neutropenia (OR, 1.59; 95 % CI, 0.34–7.54; p=0.56), anemia (OR, 0.48; 95 % CI, 0.08–2.80; p=0.41), and thrombocytopenia (OR, 0.42; 95 % CI, 0.10–1.68; p=0.22) (Figure 4). Substantial heterogeneity was observed for these outcomes (I^2^ ranged from 22 to 75 %).

**Figure 5:**
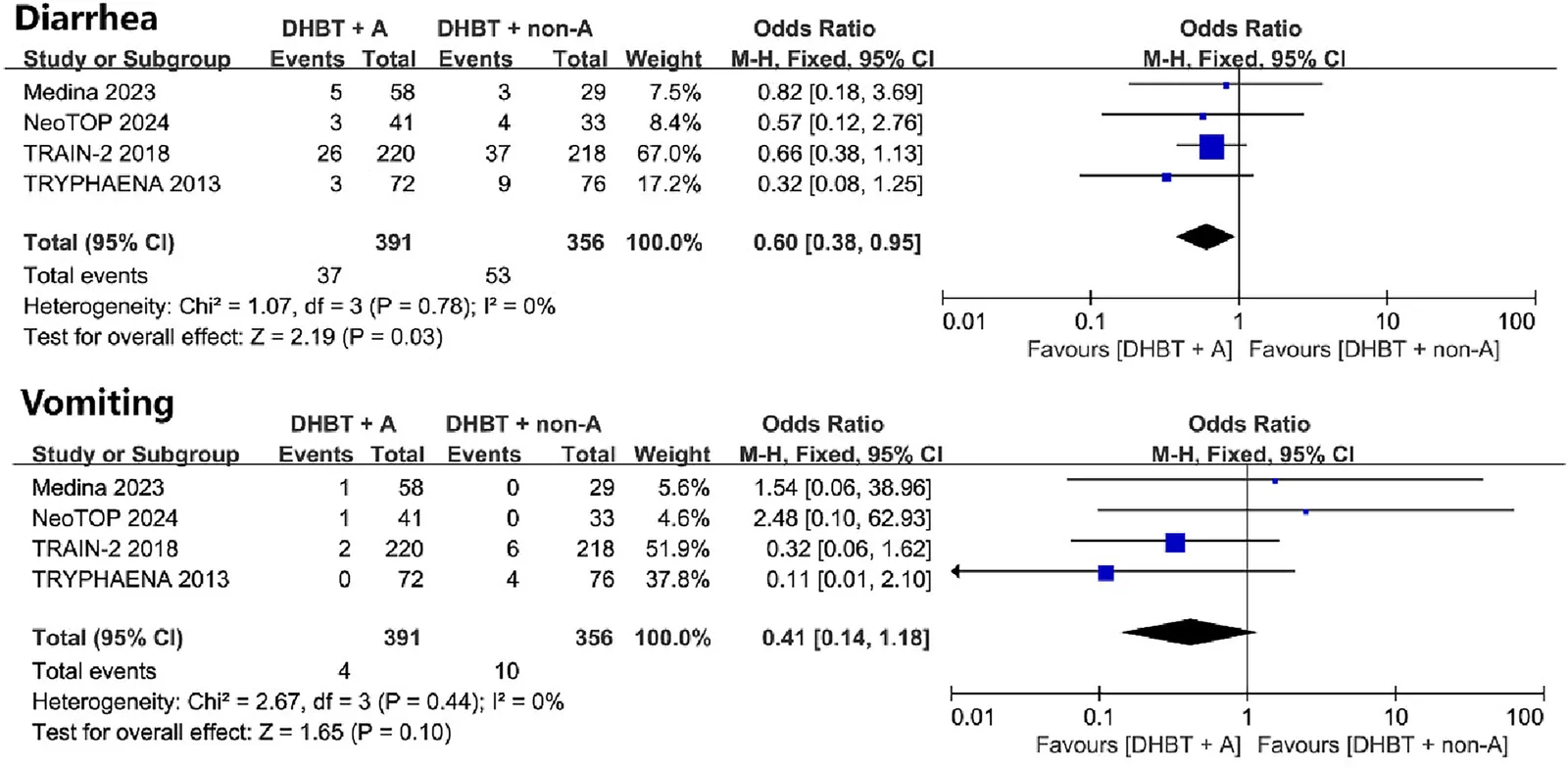
Pooled analyses of grade ≥3 non-hematological toxicity when comparing DHBT + A vs. DHBT + non-A. According to the available data for comparative analysis, diarrhea and vomiting were each available in five studies.

For non-hematological toxicities, the non-A group had a significantly higher incidence of grade ≥3 diarrhea compared with the A-based group (OR, 0.60; 95 % CI, 0.38–0.95; p=0.03), with no observed heterogeneity (I^2^=0 %). The incidence of grade ≥3 vomiting did not differ significantly between groups (Figure 5).

### Sensitivity analysis and publication bias

The leave-one-out sensitivity analysis demonstrated that the pooled estimates for all outcomes with I^2^≥30 % retained the same direction and statistical non-significance across all leave-one-out iterations; for the composite DFS/EFS/PFS endpoint, however, the magnitude of the effect estimate was sensitive to the omission of individual studies (Supplementary Table S10). Funnel plot was generated to visually assess publication bias. However, the interpretability was limited by the relatively small number of included studies (Supplementary Figure S3).

## Discussion

Neoadjuvant chemotherapy combined with dual HER2 blockade has significantly improved pCR rates and long-term survival in patients with HER2-positive early breast cancer, establishing this approach as a standard treatment [2], [3], [4], [5], [6], [7], [8]. However, optimizing the chemotherapy regimen within this framework to maintain efficacy while minimizing toxicity, especially in clarifying the role of anthracyclines, remains a critical unresolved issue. This meta-analysis systematically evaluates the efficacy and safety of A-based vs. non-A regimens combined with DHBT, aiming to provide high-level evidence to inform this clinical decision.

This meta-analysis demonstrates that omitting anthracyclines from dual HER2 blockade-based neoadjuvant therapy is not associated with a significant reduction in pathological complete response (pCR) rates. However, caution is warranted when extrapolating these findings to long-term survival outcomes. While funnel plots were employed to assess publication bias, their interpretability is constrained by the relatively limited number of included studies – particularly regarding survival endpoints – which undermines the statistical power to detect asymmetry. Although our pooled estimates revealed no statistically significant detriment in survival, these findings align with pivotal phase II/III trials such as TRAIN-2 and TRYPHAENA [10], 11], 14], 15], supporting non-anthracycline regimens as an evidence-based option in the contemporary neoadjuvant landscape. Nevertheless, the current evidence base remains insufficient to definitively establish long-term non-inferiority. Furthermore, potent HER2 pathway inhibition may partially offset the requirement for anthracycline-based cytotoxicity in many patients.

Although anthracycline-related cardiotoxicity remains a central concern in clinical practice, this meta-analysis did not reveal significant differences in cardiac outcomes between the treatment groups. This finding should, however, be interpreted with caution. First, the follow-up periods in the included studies were relatively short. For instance, Medina et al. primarily covered the neoadjuvant phase [22], and the TRAIN-2 trial also did not fully assess the potential long-term cardiac risks associated with anthracycline use [10]. Second, substantial heterogeneity (I^2^>50 %) was observed among cardiac outcomes, likely due to variations in definitions of cardiac events, monitoring protocols, and baseline cardiovascular risk profiles of enrolled patients. Therefore, the current evidence suggests no clear increase in short-term cardiotoxicity when anthracyclines are added to a dual HER2-targeted neoadjuvant regimen, but it does not establish equivalent long-term cardiac safety compared with non-A regimens. In clinical practice, patients receiving A-based therapy should still undergo guideline-recommended baseline assessment and regular cardiac monitoring. Novel formulations such as liposomal doxorubicin may offer improved safety and warrant further investigation in future studies.

This study identified substantial heterogeneity (I^2^≥50 %) in the composite progression-free survival endpoints (DFS/EFS/PFS) and in certain hematological toxicity indices. Notably, the aggregation of these distinct endpoints, despite increasing statistical precision, is a major source of the high heterogeneity (I^2^=78 %) due to fundamental differences in their definitions. Specifically, DFS (adjuvant recurrence/death), EFS (broader event capture including treatment cessation), and PFS (radiological progression in metastatic settings) are not clinically interchangeable. Variability in event definitions (e.g., inclusion of second cancers or treatment-related mortality) across included trials introduced measurement bias, substantially contributing to the I^2^ value. Additionally, residual confounding arose from heterogeneity within chemotherapy classes (agent selection, dose intensity, G-CSF utilization), biological diversity within HER2-positive disease (HR status, Ki-67), and methodological variations in trial conduct (eligibility criteria, follow-up duration). To address this, future studies should perform subgroup analyses based on specific endpoint definitions to ensure robustness.

A notable finding of this meta-analysis is the significantly higher risk of grade ≥3 diarrhea in the non-A group, predominantly represented by TCbHP-based regimens, compared with the A-based group (OR, 0.60; p=0.03). This signal is biologically plausible because pertuzumab is associated with gastrointestinal toxicity and may add to the diarrhea risk of taxane-platinum chemotherapy. The clinical implications are twofold. First, enhanced supportive care is essential: patients receiving TCbHP should receive pretreatment education, early access to antidiarrheal agents such as loperamide, and close monitoring of hydration, electrolytes, and treatment adherence. Temporary treatment interruption and chemotherapy dose modification may be warranted in severe or persistent cases. Second, this toxicity profile should be considered during regimen selection. When expected efficacy is similar, the higher incidence of severe diarrhea with TCbHP may influence treatment choice, particularly for older patients or those with baseline gastrointestinal vulnerability. However, the interpretation of these toxicity data is subject to methodological variability across studies. As detailed in Supplementary Table S9, differences in toxicity grading criteria (e.g., CTCAE versions) and, where reported, supportive care protocols (e.g., G-CSF prophylaxis) may have contributed to the observed heterogeneity. Notably, supportive care strategies were not reported in several of the included trials, so this attribution remains partly inferential and reinforces the need for standardized reporting in future trials.

Although dual HER2 blockade plus anthracyclines showed no population-level efficacy gain, the pooled data cannot exclude selective benefit in high-risk subgroups. Notably, the KRISTINE trial demonstrated that chemotherapy-sparing approaches may increase preoperative progression (3-year EFS 85.3 vs. 94.2 % with TCH + P) [13]. Supporting this, retrospective data link anthracycline inclusion to higher event-free survival in inflammatory breast cancer [16]. Mechanistically, TOP2A amplification (present in 30–40 % of HER2+ tumors) predicts sensitivity to topoisomerase II inhibitors, unlike CEP17 polysomy [25], aligning with the 2023 St Gallen recommendation for anthracycline retention in high-risk disease [26]. Pegylated liposomal doxorubicin (PLD) offers a potential mitigation strategy, achieving tpCR of 60.3 % overall and >63 % in node-positive/stage III disease with minimal cardiotoxicity [27]. However, these findings remain hypothesis-generating due to the lack of individual patient data and pre-specified subgroup analyses in the included RCTs. Future trials should prioritize biomarker-driven enrichment to validate these observations.

This imperative intensifies as de-escalation evolves beyond the “anthracycline vs. non-anthracycline” dichotomy. First-generation refinements remained confined to the cytotoxic paradigm – substituting epirubicin for doxorubicin or adopting pegylated liposomal doxorubicin (PLD). Second-generation strategies pursued chemo-sparing via anthracycline omission and treatment shortening while retaining a cytotoxic backbone – exemplified by neoCARHP (THP vs. TCbHP; noninferior pCR 64.1 vs. 65.9 %) [28], the PET-adaptive PHERGain platform (3-y iDFS 94.8 % overall; 96.4 % in chemo-spared pCR subgroup) [29], and WSG-ADAPT-TP, which delineated a chemo-free niche for HR+/HER2+ disease (5-y iDFS 92.7 % in pCR subgroup; 92.1 % when ACT omitted) [30] – none displaced chemotherapy entirely. The emerging third generation now leverages HER2-directed ADCs to displace, rather than merely modulate, this foundation. The phase III DESTINY-Breast11 trial crystallizes this shift: in high-risk HER2-positive early breast cancer, neoadjuvant T-DXd-THP significantly improved pCR vs. ddAC-THP (67.3 vs. 56.3 %; p=0.003), with superior RCB-0/I rates (81.3 vs. 69.1 %) and fewer grade ≥3 adverse events (37.5 vs. 55.8 %) [31]. Notably, the pronounced pCR benefit in HR-negative tumors (83.1 vs. 67.1 %) upends the longstanding assumption that this subset requires anthracyclines [31].

These advances are consistent with evolving guidelines: the 2023 St. Gallen Consensus restricts anthracycline/PLD use to IBC, N2–3 disease, or TOP2A-amplified tumors [26], while the 2025–2026 NCCN Guidelines designate T-DXd-based neoadjuvant therapy as an emerging option [8], 32], advancing the move toward biomarker-guided de-escalation. Nevertheless, contemporary ADC trials currently lack pre-specified TOP2A or other biomarker subgroup analyses to refine these indications. Addressing this knowledge gap – identifying biomarkers beyond HR status to guide chemotherapy omission – remains a critical priority for future trial design as the field shifts from “anthracycline-by-default” to biomarker-informed, risk-adapted de-escalation.

This study exclusively analyzed prospective clinical studies using dual HER2 blockade, providing more clinically relevant evidence than previous meta-analyses that included single HER2 blockade or retrospective data [18]. Our findings are consistent with the current use of non-A dual-targeted regimens for many patients with HER2-positive breast cancer, while emphasizing that regimen selection should account for toxicity profile, cardiovascular risk, tumor burden, and patient preferences. Future research should focus on three directions: (1) precision patient selection, integrating biomarkers such as TOP2A status, hormone receptor expression, and refined molecular subtypes to determine whether any subgroup derives additional benefit from A-based therapy; Beyond static genomic alterations such as TOP2A, emerging evidence underscores the predictive utility of metabolic-immune remodeling and the tumor microenvironment (TME) [33], 34]. Integrating multi-omics profiling into predictive algorithms promises to transcend the constraints of conventional clinicopathological parameters and refine patient selection. (2) novel therapeutic strategies, including antibody-drug conjugates such as T-DXd, bispecific antibodies, and chemo-light or chemotherapy-free approaches tested against optimized chemotherapy backbones; and (3) AI-assisted decision-making, using machine learning models that integrate clinical, pathological, imaging, and genomic data to predict individual efficacy and toxicity risks and support treatment personalization.

## Conclusions

In summary, this meta-analysis indicates that omitting anthracyclines from neoadjuvant dual-targeted therapy yields comparable pCR rates in patients with HER2-positive breast cancer. However, caution is warranted when interpreting survival outcomes: while no significant detriment was observed, these estimates are derived from a limited number of studies with constrained statistical power, precluding definitive conclusions regarding long-term efficacy. Therefore, the noninferiority of non-anthracycline regimens concerning long-term survival remains to be validated by mature data from Phase III trials. Notably, non-anthracycline therapy was associated with a higher risk of severe diarrhea, warranting proactive clinical management. Although short-term cardiac safety appeared similar, available evidence is insufficient to exclude potential late-onset cardiotoxicity. Treatment decisions should be individualized based on tumor biology, disease burden, baseline cardiovascular risk, toxicity tolerance, and patient preferences. Future investigations must extend beyond conventional clinicopathological parameters to integrate TOP2A status, metabolic-immune remodeling, and tumor microenvironment features, leveraging AI-supported predictive tools to refine patient selection and navigate the evolving landscape of ADC-based de-escalation strategies.

## Supplementary Material

Supplementary Material
