# Supplementary material for: Neoadjuvant anthracycline-containing vs. anthracycline-free chemotherapy with dual HER2 blockade in HER2-Positive breast cancer: a Systematic Review and meta-analysis
Source: Open Med (Wars). 2026 Sep 10;21(1):20261525. doi: 10.1515/med-2026-1525 (PMC13561121; doi:10.1515/med-2026-1525)
Supplement: Supplementary file 1 [file j_med-2026-1525_suppl_001.docx]

Figure S1 Risk of Bias summary of included RCT studies

Figure S2 Risk of bias assessment of included RCT studies

Figure S3 The funnel plot of publication bias

Table S1 The search strategies for Cochrane Library database

| ID | Search details | Results |
| --- | --- | --- |
| #1 | MeSH descriptor: [Breast Neoplasms] explode all trees | 21435 |
| #2 | (HER2 positive breast cancer) OR (HER2/neu positive) OR (ERBB2 positive) | 6063 |
| #3 | #1 AND #2 | 2105 |
| #4 | ("dual HER2 blockade":ti,ab,kw OR "double targeting":ti,ab,kw) in Title, Abstract, Keywords | 110 |
| #5 | ( (trastuzumab OR pertuzumab OR lapatinib OR neratinib OR tucatinib OR pyrotinib OR "T-DM1" OR "fam-trastuzumab deruxtecan"):ti,ab,kw ) in Title, Abstract, Keywords | 3401 |
| #6 | MeSH descriptor: [Antibodies, Monoclonal, Humanized] explode all trees | 17216 |
| #7 | MeSH descriptor: [Protein Kinase Inhibitors] explode all trees | 2256 |
| #8 | #4 OR #5 | 3406 |
| #9 | #6 OR #7 | 19289 |
| #10 | #8 AND #9 | 1308 |
| #11 | (anthracycline-based OR doxorubicin OR epirubicin OR daunorubicin) in Title, Abstract, Keywords | 10742 |
| #12 | (non-anthracycline OR taxane OR docetaxel OR paclitaxel OR carboplatin OR cyclophosphamide) in Title, Abstract, Keywords | 29403 |
| #13 | #3 OR #10 OR #11 OR #12 | 35372 |
| #14 | MeSH descriptor: [Neoadjuvant Therapy] explode all trees | 2915 |
| #15 | (neoadjuvant therapy) OR (preoperative chemotherapy) OR (pathologic complete response) OR (pCR) in Title, Abstract, Keywords | 26597 |
| #16 | (efficacy OR safety OR survival OR adverse events OR cardiotoxicity) in Title, Abstract, Keywords | 598849 |
| #17 | #14 OR #15 OR #16 | 16610992 |
| #18 | #3 AND #13 AND #17 | 1818 |

Table S2 The search strategies for Pubmed database

| ID | Search details | Results |
| --- | --- | --- |
| #1 | "Breast Neoplasms"[MeSH Terms] OR "receptor, erbb 2"[MeSH Terms] | 389784 |
| #2 | "HER2-positive breast cancer"[Title/Abstract] OR "ERBB2"[Title/Abstract] | 4416 |
| #3 | #1 OR #2 | 391091 |
| #4 | "antibodies, monoclonal, humanized"[MeSH Terms] OR "Protein Kinase Inhibitors"[MeSH Terms] | 173550 |
| #5 | "Antibody-Drug Conjugates"[Title/Abstract] | 6075 |
| #6 | "Dual HER2 blockade"[Title/Abstract] OR "Dual targeting"[Title/Abstract] OR "dual HER2 inhibition"[Title/Abstract] OR "Trastuzumab"[Title/Abstract] OR "Herceptin"[Title/Abstract] OR "Pertuzumab"[Title/Abstract] OR "Lapatinib"[Title/Abstract] OR "Neratinib"[Title/Abstract] OR "Tucatinib"[Title/Abstract] OR "Pyrotinib"[Title/Abstract] OR "Trastuzumab emtansine"[Title/Abstract] OR "T-DM1"[Title/Abstract] OR "Fam-trastuzumab deruxtecan"[Title/Abstract] OR "DS-8201"[Title/Abstract] | 22827 |
| #7 | #4 OR #5 OR #6 | 190655 |
| #8 | #3 AND #7 | 18698 |
| #9 | "Anthracyclines"[MeSH Terms] OR "Taxoids"[MeSH Terms] OR "Antineoplastic Combined Chemotherapy Protocols"[MeSH Terms] | 258113 |
| #10 | "Doxorubicin"[Title/Abstract] OR "Epirubicin"[Title/Abstract] OR "anthracycline-based"[Title/Abstract] OR "non-anthracycline"[Title/Abstract] OR "Docetaxel"[Title/Abstract] OR "Paclitaxel"[Title/Abstract] OR "Carboplatin"[Title/Abstract] OR "Cyclophosphamide"[Title/Abstract] | 180446 |
| #11 | #9 OR #10 | 344428 |
| #12 | #8 AND #11 | 6463 |
| #13 | "Neoadjuvant Therapy"[MeSH Terms] OR "Treatment Outcome"[MeSH Terms] OR "Drug-Related Side Effects and Adverse Reactions"[MeSH Terms] | 1546720 |
| #14 | "neoadjuvant therapy"[Title/Abstract] OR "preoperative chemotherapy"[Title/Abstract] OR "pathologic complete response"[Title/Abstract] OR "pCR"[Title/Abstract] OR "survival"[Title/Abstract] OR "cardiotoxicity"[Title/Abstract] OR "diarrhea"[Title/Abstract] OR "neutropenia"[Title/Abstract] | 2179795 |
| #15 | #13 OR #14 | 3459979 |
| #16 | #12 AND #15 | 4494 |
| #17 | #16 AND 2010/01/01:2026/02/06[Date - Publication] | 3837 |

Table S3 The search strategies for EMBASE database

| ID | Search details | Results |
| --- | --- | --- |
| #1 | 'breast tumor'/exp OR 'human epidermal growth factor receptor 2 positive breast cancer'/exp | 800110 |
| #2 | 'her2 overexpressing breast cancer':ti,ab,kw | 783 |
| #3 | 'erbb2 positive':ti,ab,kw | 692 |
| #4 | #1 OR #2 OR #3 | 800343 |
| #5 | 'trastuzumab'/exp OR 'pertuzumab'/exp OR 'lapatinib'/exp OR 'neratinib'/exp OR 'tucatinib'/exp OR 'pyrotinib'/exp OR 'trastuzumab emtansine'/exp OR 'trastuzumab deruxtecan'/exp | 71320 |
| #6 | 'dual her2 blockade':ti,ab,kw OR 'double targeting therapy':ti,ab,kw OR 'dual anti-her2':ti,ab,kw | 691 |
| #7 | #5 OR #6 | 71405 |
| #8 | 'anthracycline based chemotherapy' OR 'doxorubicin'/exp OR 'epirubicin'/exp OR 'taxane'/exp OR 'carboplatin'/exp OR 'cyclophosphamide'/exp | 526218 |
| #9 | 'non-anthracycline regimen':ti,ab,kw | 37 |
| #10 | #8 OR #9 | 526234 |
| #11 | 'neoadjuvant therapy'/exp OR 'treatment outcome'/exp OR 'adverse drug reaction'/exp OR 'cardiotoxicity'/exp OR 'diarrhea'/exp | 4218701 |
| #12 | 'preoperative chemotherapy':ti,ab,kw OR 'pathologic complete response':ti,ab,kw OR 'pcr':ti,ab,kw | 1017535 |
| #13 | #11 OR #12 | 5158555 |
| #14 | [2010-01-01]/sd | 26548286 |
| #15 | #4 AND #7 AND #10 AND #13 AND #14 | 7013 |

Table S4 PRISMA_2020_checklist

| **Section and Topic** | **Item #** | **Checklist item** | **Reported on page#** |
| --- | --- | --- | --- |
| **TITLE** | | |  |
| Title | 1 | Identify the report as a systematic review. | 1 |
| **ABSTRACT** | | |  |
| Abstract | 2 | See the PRISMA 2020 for Abstracts checklist. | 1 |
| **INTRODUCTION** | | |  |
| Rationale | 3 | Describe the rationale for the review in the context of existing knowledge. | 2-3 |
| Objectives | 4 | Provide an explicit statement of the objective(s) or question(s) the review addresses. | 3 |
| **METHODS** | | |  |
| Eligibility criteria | 5 | Specify the inclusion and exclusion criteria for the review and how studies were grouped for the syntheses. | 3-4 |
| Information sources | 6 | Specify all databases, registers, websites, organisations, reference lists and other sources searched or consulted to identify studies. Specify the date when each source was last searched or consulted. | 3 |
| Search strategy | 7 | Present the full search strategies for all databases, registers and websites, including any filters and limits used. | 3 |
| Selection process | 8 | Specify the methods used to decide whether a study met the inclusion criteria of the review, including how many reviewers screened each record and each report retrieved, whether they worked independently, and if applicable, details of automation tools used in the process. | 3-4 |
| Data collection process | 9 | Specify the methods used to collect data from reports, including how many reviewers collected data from each report, whether they worked independently, any processes for obtaining or confirming data from study investigators, and if applicable, details of automation tools used in the process. | 3-4 |
| Data items | 10a | List and define all outcomes for which data were sought. Specify whether all results that were compatible with each outcome domain in each study were sought (e.g. for all measures, time points, analyses), and if not, the methods used to decide which results to collect. | 4 |
|  | 10b | List and define all other variables for which data were sought (e.g. participant and intervention characteristics, funding sources). Describe any assumptions made about any missing or unclear information. | 4 |
| Study risk of bias assessment | 11 | Specify the methods used to assess risk of bias in the included studies, including details of the tool(s) used, how many reviewers assessed each study and whether they worked independently, and if applicable, details of automation tools used in the process. | 4 |
| Effect measures | 12 | Specify for each outcome the effect measure(s) (e.g. risk ratio, mean difference) used in the synthesis or presentation of results. | 4 |
| Synthesis methods | 13a | Describe the processes used to decide which studies were eligible for each synthesis (e.g. tabulating the study intervention characteristics and comparing against the planned groups for each synthesis (item #5)). | 4 |
|  | 13b | Describe any methods required to prepare the data for presentation or synthesis, such as handling of missing summary statistics, or data conversions. | 4 |
|  | 13c | Describe any methods used to tabulate or visually display results of individual studies and syntheses. | 4 |
|  | 13d | Describe any methods used to synthesize results and provide a rationale for the choice(s). If meta-analysis was performed, describe the model(s), method(s) to identify the presence and extent of statistical heterogeneity, and software package(s) used. | 4 |
|  | 13e | Describe any methods used to explore possible causes of heterogeneity among study results (e.g. subgroup analysis, meta-regression). | 5 |
|  | 13f | Describe any sensitivity analyses conducted to assess robustness of the synthesized results. | 5 |
| Reporting bias assessment | 14 | Describe any methods used to assess risk of bias due to missing results in a synthesis (arising from reporting biases). | 5 |
| Certainty assessment | 15 | Describe any methods used to assess certainty (or confidence) in the body of evidence for an outcome. | 4-5 |
| **RESULTS** | | |  |
| Study selection | 16a | Describe the results of the search and selection process, from the number of records identified in the search to the number of studies included in the review, ideally using a flow diagram. | 5 |
|  | 16b | Cite studies that might appear to meet the inclusion criteria, but which were excluded, and explain why they were excluded. | 5-6 |
| Study characteristics | 17 | Cite each included study and present its characteristics. | 7-9 |
| Risk of bias in studies | 18 | Present assessments of risk of bias for each included study. | 4-5 |
| Results of individual studies | 19 | For all outcomes, present, for each study: (a) summary statistics for each group (where appropriate) and (b) an effect estimate and its precision (e.g. confidence/credible interval), ideally using structured tables or plots. | 10-13 |
| Results of syntheses | 20a | For each synthesis, briefly summarise the characteristics and risk of bias among contributing studies. | 10-13 |
|  | 20b | Present results of all statistical syntheses conducted. If meta-analysis was done, present for each the summary estimate and its precision (e.g. confidence/credible interval) and measures of statistical heterogeneity. If comparing groups, describe the direction of the effect. | 10-13 |
|  | 20c | Present results of all investigations of possible causes of heterogeneity among study results. | 10-13 |
|  | 20d | Present results of all sensitivity analyses conducted to assess the robustness of the synthesized results. | 13 |
| Reporting biases | 21 | Present assessments of risk of bias due to missing results (arising from reporting biases) for each synthesis assessed. | 13 |
| Certainty of evidence | 22 | Present assessments of certainty (or confidence) in the body of evidence for each outcome assessed. | 13 |
| **DISCUSSION** | | |  |
| Discussion | 23a | Provide a general interpretation of the results in the context of other evidence. | 13 |
|  | 23b | Discuss any limitations of the evidence included in the review. | 14-15 |
|  | 23c | Discuss any limitations of the review processes used. | 14-15 |
|  | 23d | Discuss implications of the results for practice, policy, and future research. | 15-16 |
| **OTHER INFORMATION** | | |  |
| Registration and protocol | 24a | Provide registration information for the review, including register name and registration number, or state that the review was not registered. | 3 |
|  | 24b | Indicate where the review protocol can be accessed, or state that a protocol was not prepared. | 3 |
|  | 24c | Describe and explain any amendments to information provided at registration or in the protocol. | 3 |
| Support | 25 | Describe sources of financial or non-financial support for the review, and the role of the funders or sponsors in the review. | 17 |
| Competing interests | 26 | Declare any competing interests of review authors. | 17 |
| Availability of data, code and other materials | 27 | Report which of the following are publicly available and where they can be found: template data collection forms; data extracted from included studies; data used for all analyses; analytic code; any other materials used in the review. | 17 |

*From:* Page MJ, McKenzie JE, Bossuyt PM, Boutron I, Hoffmann TC, Mulrow CD, et al. The PRISMA 2020 statement: an updated guideline for reporting systematic reviews. BMJ 2021;372:n71. doi: 10.1136/bmj.n71. This work is licensed under CC BY 4.0. To view a copy of this license, visit <https://creativecommons.org/licenses/by/4.0/>

Table S5 MOOSE checklist

| **Reporting of background should include** | |
| --- | --- |
| Problem definition | P2 |
| Hypothesis statement | P2-3 |
| Description of study outcome(s) | P4 |
| Type of exposure or intervention used | P4 |
| Type of study designs used | P4 |
| Study population | P3 |
| **Reporting of search strategy should include** | |
| Qualifications of searchers (eg, librarians and investigators) | P3 |
| Search strategy, including time period included in the synthesis and keywords | P3-4 |
| Effort to include all available studies, including contact with authors | P3-4 |
| Databases and registries searched | P3 |
| Search software used, name and version, including special features used (eg, explosion) | P3-5 |
| Use of hand searching (eg, reference lists of obtained articles) | P3-4 |
| List of citations located and those excluded, including justification | P4 |
| Method of addressing articles published in languages other than English | P3-4, 6 |
| Method of handling abstracts and unpublished studies | P4 |
| Description of any contact with authors | P4 |
| **Reporting of methods should include** | |
| Description of relevance or appropriateness of studies assembled for assessing the hypothesis to be tested | P4-5 |
| Rationale for the selection and coding of data (eg, sound clinical principles or convenience) | P4-5 |
| Documentation of how data were classified and coded (eg, multiple raters, blinding, and interrater reliability) | P4-5 |
| Assessment of confounding (eg, comparability of cases and controls in studies where appropriate) | P4-5 |
| Assessment of study quality, including blinding of quality assessors; stratification or regression on possible predictors of study results | P4-5 |
| Assessment of heterogeneity | P5 |
| Description of statistical methods (eg, complete description of fixed or random effects models, justification of whether the chosen models account for predictors of study results, dose-response models, or cumulative meta-analysis) in sufficient detail to be replicated | P5 |
| Provision of appropriate tables and graphics | P6-13 |
| **Reporting of results should include** | |
| Graphic summarizing individual study estimates and overall estimate | P7-9 |
| Table giving descriptive information for each study included | P7-9 |
| Results of sensitivity testing (eg, subgroup analysis) | P13 |
| Indication of statistical uncertainty of findings | P10-13 |
| **Reporting of discussion should include** | |
| Quantitative assessment of bias (eg, publication bias) | P13-16 |
| Justification for exclusion (eg, exclusion of non-English-language citations) | P13-16 |
| Assessment of quality of included studies | P13-16 |
| **Reporting of conclusions should include** | |
| Consideration of alternative explanations for observed results | P15-16 |
| Generalisation of the conclusions (ie, appropriate for the data presented and within the domain of the literature review) | P15-16 |
| Guidelines for future research | P16-17 |
| Disclosure of funding source | P17 |

Table S6. The quality assessment of non-RCTs using MINORS score.

| Study | A clearly stated aim | Inclusion of consecutive patients | Prospective collection of data | Endpoints appropriate to the objective of the study | Unbiased assessment of the study endpoint | Follow-up period appropriate to the objective of the study | Loss to follow up less than 5% | Prospective calculation of the study size | Additional criteria in the case of comparative study | | | |
| --- | --- | --- | --- | --- | --- | --- | --- | --- | --- | --- | --- | --- |
|  |  |  |  |  |  |  |  |  | An adequate control group | Contemporary groups | Baseline equivalence of groups | Adequate statistical analyses |
| NeoTOP 2024 | 2 | 2 | 2 | 2 | 1 | 1 | 0 | 0 | 2 | 2 | 1 | 1 |

*The items are scored 0 (not reported), 1 (reported but inadequate) or 2 (reported and adequate). The global ideal score is 24 for comparative studies.

Table S7. The quality assessment of cohort studies using the NOS assessment scale.

| Study | Selection | | | | Comparability | | Outcome | | | Total number of stars |
| --- | --- | --- | --- | --- | --- | --- | --- | --- | --- | --- |
|  | Representativeness of the exposed cohort | Selection of the non exposed cohort | Ascertainment of exposure | Demonstration that outcome of interest was not present at start of study | | Comparability of cohorts on the basis of the design or analysis | Assessment of outcome | Was follow-up long enough for outcomes to occur | Adequacy of follow up of cohorts |  |
| Medina 2023 | ★ | ★ | ★ | ★ | | ★★ | ★ | ★ | - | 8 |
| Alonso-Romero  2026 | ★ | ★ | ★ | ★ | | ★ | ★ | - | ★ | 7 |

* According to the total number of stars, ≥7 stars were considered to be high quality.

Table S8. The definitions of pCR, DFS, EFS, PFS, OS and CAEs in different studies

| Study | The definition of pCR | The definitions of DFS/EFS/PFS | The definition of OS | The definition of CAEs |
| --- | --- | --- | --- | --- |
| TRYPHAENA [14-15] | pCR was assessed locally at the time of surgery and deﬁned as the absence of invasive neoplastic cells during microscopic assessment of the primary tumour (bpCR; ypT0/is) or in the breast and axilla (tpCR; ypT0/Tis, ypN0). | DFS, deﬁned as the time from the ﬁrst date of no disease i.e. date of surgery, to the ﬁrst documentation of  progressive disease. | OS, deﬁned as the time from randomisation to death from any cause. | Incidence of symptomatic left ventricular systolic dysfunction (LVSD) as assessed by the investigator, and LVEF decline ≥10% points from baseline to <50% over the course of neoadjuvant treatment. |
| TRAIN-2 [10-11] | pCR, defined as the absence of invasive tumour cells in the breast and axilla (ypT0/is ypN0). | EFS, defined as the time from randomization to disease progression resulting in inoperability, recurrence (contralateral ductal carcinoma in situ excluded), secondary primary malignant neo-  plasms, or death by any cause (equal to progression-free  survival). | OS, deﬁned as the time from randomisation to death from any cause. | A decline in LVEF of 10% or more from baseline to less than 50%. |
| Medina 2023[22] | pCR, defined as the absence of invasive carcinoma in both the breast and lymph nodes (ypT0 ypN0 or ypTis ypN0). | NA | NA | Reduction in LVEF. |
| NeoTOP 2024[23] | pCR was defined by the absence of invasive cancer cells in both breast tissues and lymph nodes. | PFS, defined as the time from the first administration of treatment to progression or death from any cause (if progression had not been documented). | OS, defined as the time from the first  administration of treatment to death from any cause. | NA |
| Alonso-Romero  2026[24] | pCR. defined as the absence of invasive tumor in both the breast and axillary lymph nodes (ypT0/ypTis and ypN0). | NA | NA | NA |

Abbreviations: pCR, pathological complete response; DFS, disease-free survival; PFS, progression-free survival; OS, overall survival; EFS, event-free survival; CAEs, cardiac adverse events; LVEF, left ventricular ejection fraction; NA, not applicable.

Table S9. Details regarding differences in chemotherapy backbone, supportive care strategies, and toxicity assessment methods across studies.

| Study | Details regarding differences in chemotherapy backbone | Supportive care strategies | Toxicity assessment methods |
| --- | --- | --- | --- |
| TRYPHAENA [14-15] | group A (cycles 1-6: trastuzumab [8 mg/kg loading dose and 6 mg/kg maintenance] plus pertuzumab [840 mg loading dose and 420 mg maintenance], plus 5-fluorouracil, epirubicin and cyclophosphamide [FEC] [cycles 1-3; 500 mg/m^2^ 5-fluorouracil/100 mg/m^2^ epirubicin/600 mg/m^2^ cyclophosphamide] then docetaxel [cycles 4-6; 75 mg/m^2^, escalated to 100 mg/m^2^ if well tolerated]), B (cycles 1-3: FEC, cycles 4-6: trastuzumab plus pertuzumab plus docetaxel as mentioned previously) or C (cycles 1-6: trastuzumab plus pertuzumab plus docetaxel [75 mg/m^2^, without dose escalation], and carboplatin [AUC 6]). | Not reported in the paper. | Adverse events were monitored continuously and graded according to the National Cancer Institute Common Terminology Criteria for Adverse Events, version 3.0.  Symptomatic LVSD was reported as a serious adverse event (SAE). |
| TRAIN-2 [11-12] | Group A: 5-fluorouracil (500 mg/m²), epirubicin (90 mg/m²), and cyclophosphamide (500 mg/m²) every 3 weeks for three cycles followed by paclitaxel (80 mg/m² on days 1 and 8) and carboplatin (area under the concentration-time curve [AUC] 6 mg/mL per min on day 1 or optionally, as per hospital preference, AUC 3 mg/mL per min on days 1 and 8) every 3 weeks for six cycles,  Group B: 9 cycles of paclitaxel and carboplatin at the same dose and schedule as in the anthracycline group.  Patients in both study groups received trastuzumab (6 mg/kg, loading dose 8 mg/kg) and pertuzumab (420 mg, loading dose 840 mg) concurrently with all chemotherapy cycles. | 1. Secondary granulocyte-colony-stimulating factor (G-CSF) prophylaxis was initiated.  2. Based on the grading of adverse events, adjust the dosage of chemotherapy drugs when necessary, and even temporarily or permanently discontinue the treatment.  3. Primary and secondary prevention for diarrhea. | Adverse events were assessed with each cycle of treatment according to Common Terminology Criteria for Adverse Events (CTCAE) version 4.03. |
| Medina 2023[22] | DPT [docetaxel-IV pertuzumab- SC trastuzumab 600 mg; 4 cycles]: docetaxel (IV) 100 mg/m^2^ and pertuzumab (IV) 840 mg on day1 of cycle 1; subsequently, pertuzumab (IV) 420 mg was administered on day 1 of cycles 2-4 and trastuzumab 600 mg (SC) was administered on day 1 cycles 1-4. The cycles were repeated every 21 days.  ddAC-DPT [dose-dense anthracycline-based scheme+DPT; 8 cycles]: doxorubicin 60 mg/m2 (IV) and cyclophosphamide 600 mg/m2 (IV) on day 1 every 14 days of cycles 1-4. It was followed sequentially by docetaxel 100 mg/m2 (IV) and pertuzumab 840 mg (IV) on day 1 of cycle 1, and then by pertuzumab 420 mg (IV) and trastuzumab 600 mg (SC) on day 1 of  cycles 2-4. The cycles were repeated every 21 days. | 1. The use of granulocyte colony-stimulating factor (G-CSF).  2. Based on the grading of adverse events, adjust the dosage of drugs when necessary, and even temporarily or permanently discontinue the treatment. | Adverse events were described and graded according to Common Terminology Criteria for Adverse Events (CTCAE) CV4. |
| NeoTOP 2024[23] | In the TOP2A + group, the anthracycline-based chemotherapy consisted of FEC 100 (5-Fluorouracil 500mg/m^2^, Epirubicin 100mg/m^2^, Cyclophosphamide 500mg/m^2^) for 3 cycles  followed by 3 cycles of Trastuzumab (8 mg/kg at cycle 4 then 6 mg/kg at cycles 5 and 6; IV), Pertuzumab (840 mg/kg at cycle 4 then 420 mg/kg at cycles 5 and 6; IV), and Docetaxel (75mg/m2 at cycle 4 then 100mg/m2 at cycles 5  and 6; IV).  In the TOP2A- group, chemotherapy consisted of 6 cycles of Docetaxel (75mg/m2; IV) and Carboplatin (target AUC 6 mg/ml/min; IV) associated with Trastuzumab (loading dose of 8 mg/kg then 6 mg/kg; IV) and Pertuzumab (loading dose of 840 mg/kg then 420 mg/kg; IV).  In both groups, Trastuzumab was continued as adjuvant treatment and patients received a total of 18 infusions. | Not reported in the paper. | Severity was graded according to the National Cancer Institute Common Terminology Criteria for Adverse Events version 4.0 (NCI CTCAE v4.0). |
| Alonso-Romero  2026[24] | Scheme 1: Adriamycin 60 mg/m2 (or epirubicin 90 mg/m^2^) + cyclophosphamide 600 mg/m^2^ × 4 cycles, every 15 or 21 days (according to the usual clinical protocol of the service), followed by paclitaxel 80 mg/m^2^ weekly × 12 weeks (or docetaxel 100 mg/m^2^ every 3 weeks × 4), pertuzumab 840 mg in cycle 1 and 420 mg in cycles 2-4 (cycles every 21 days) + trastuzumab CT-P6 8 mg/kg in cycle 1 and 6 mg/kg in cycles  2-4 (cycles every 21 days).  Scheme 2: Docetaxel 75 mg/m2 every 3 weeks × 6 cycles + carboplatin 5 or 6 AUC every 3 weeks × 6 cycles + pertuzumab 840 mg in cycle 1 and 420 mg in cycles 2-6 (cycles every 21 days) + trastuzumab CT-P6 8 mg/kg in cycle 1 and 6 mg/kg in cycles 2-6 (cycles every 21 days).  Scheme 3 : Paclitaxel 100 mg /m^2^ weekly × 8 weeks + pertuzumab 840 mg in cycle 1 and  420 mg in cycles 2-3 (cycles every 21 days) + trastuzumab CT-P6 8 mg/kg in cycle 1 and 6 mg/kg in cycles 2-3 (cycles every 21 days) (both administered together with doses 1, 4 and 7 of paclitaxel), followed by epirubicin 90 mg/m^2^ every 3 weeks + pertuzumab and trastuzumab CT-P6 IV every 3 weeks, with the same doses × 4 cycles. | Not reported in the paper. | Not reported in the paper. |

Table S10. Sensitivity analysis

| Items | Study omitted | Estimate | 95%CI | |
| --- | --- | --- | --- | --- |
| DFS/EFS/PFS | TRYPHAENA 2018 (DFS) | -0.886 | -2.483 | 0.712 |
|  | TRAIN-2 2021 (EFS) | -1.032 | -2.446 | 0.382 |
|  | NeoTOP 2024 (PFS) | -0.155 | -0.663 | 0.353 |
|  | Combined | -0.687 | -1.698 | 0.323 |
| CAEs | TRAIN-2 2021 | 0.572 | 0.266 | 1.231 |
|  | TRYPHAENA 2018 | 0.965 | 0.106 | 8.826 |
|  | Medina 2023 | 1.159 | 0.373 | 3.603 |
|  | Combined | 0.955 | 0.313 | 2.913 |
| LVEF decline ≥10% points from baseline to <50% | TRYPHAENA 2018 | 1.974 | 0.862 | 4.518 |
|  | TRAIN-2 2021 | 0.775 | 0.301 | 2.000 |
|  | Medina 2023 | 1.655 | 0.860 | 3.185 |
|  | Combined | 1.473 | 0.785 | 2.767 |
| Febrile neutropenia | TRAIN-2 2018 | 0.855 | 0.200 | 3.652 |
|  | Medina 2023 | 1.292 | 0.175 | 9.535 |
|  | NeoTOP 2024 | 2.758 | 0.617 | 12.340 |
|  | TRYPHAENA 2013 | 1.623 | 0.139 | 18.965 |
|  | Combined | 1.589 | 0.335 | 7.540 |
| Anaemia | TRAIN-2 2018 | 0.340 | 0.019 | 6.064 |
|  | Medina 2023 | 0.244 | 0.031 | 1.950 |
|  | NeoTOP 2024 | 0.672 | 0.086 | 5.244 |
|  | TRYPHAENA 2013 | 0.926 | 0.178 | 4.825 |
|  | Combined | 0.476 | 0.081 | 2.798 |
| Thrombocytopenia | TRAIN-2 2018 | 0.208 | 0.035 | 1.234 |
|  | Medina 2023 | 0.296 | 0.050 | 1.764 |
|  | NeoTOP 2024 | 0.400 | 0.084 | 2.962 |
|  | TRYPHAENA 2013 | 0.826 | 0.517 | 1.321 |
|  | Combined | 0.420 | 0.105 | 1.685 |
